# The mortality of patients with Parkinson's disease with deep brain stimulation

**DOI:** 10.3389/fneur.2022.1099862

**Published:** 2023-01-16

**Authors:** Ahro Kim, Han-Joon Kim, Aryun Kim, Yoon Kim, Ahwon Kim, Jed Noel A. Ong, Hye Ran Park, Sun Ha Paek, Beomseok Jeon

**Affiliations:** ^1^Department of Neurology, Ulsan University Hospital, University of Ulsan College of Medicine, Ulsan, Republic of Korea; ^2^Department of Neurology, College of Medicine, Seoul National University Hospital, Seoul, Republic of Korea; ^3^Department of Neurology, Chungbuk National University Hospital, Cheongju, Republic of Korea; ^4^Department of Neurology, Young Tong Hyo Hospital, Suwon, Republic of Korea; ^5^Department of Neurosciences, Makati Medical Center, Makati, Philippines; ^6^Department of Neurosurgery, Soonchunhyang University Seoul Hospital, Seoul, Republic of Korea; ^7^Department of Neurosurgery, College of Medicine, Seoul National University Hospital, Seoul, Republic of Korea

**Keywords:** mortality, Parkinson's disease, STN (subthalamic nucleus), outcome, deep brain stimulation

## Abstract

**Background:**

Deep brain stimulation (DBS) of the subthalamic nucleus (STN) is effective in improving motor function in patients with Parkinson's disease (PD). This study aimed to investigate mortality associated with bilateral STN DBS in patients with PD and to assess the factors associated with mortality and causes of death after DBS.

**Methods:**

We reviewed the medical records of 257 patients with PD who underwent bilateral STN DBS at the Movement Disorder Center at Seoul National University Hospital between March 2005 and November 2018. Patients were evaluated preoperatively, at 3, 6, and 12 months after surgery and annually thereafter. The cause and date of death were obtained from interviews with caregivers or from medical certificates at the last follow-up.

**Results:**

Of the 257 patients with PD, 48 patients (18.7%) died, with a median time of death of 11.2 years after surgery. Pneumonia was the most common cause of death. Older age of disease onset, preoperative falling score while on medication, and higher preoperative total levodopa equivalent daily dose were associated with a higher risk of mortality in time-dependent Cox regression analysis.

**Conclusion:**

These results confirm the mortality outcome of STN DBS in patients with advanced PD.

## Introduction

Parkinson's disease (PD) is one of the most common neurodegenerative diseases worldwide. Deep brain stimulation (DBS) of the subthalamic nucleus (STN) is effective in improving motor function in patients with PD (1, 2). This effect of DBS is known to persist for at least 5–10 years, although the effect decreases as axial signs appear, with the progression of PD (3–7). It has been recently reported that STN DBS remains effective at treating motor complications 15 years after surgery (8). Long-term outcomes of DBS, with a focus on mortality, have been reported in previous studies from various countries (9–12). The mortality outcomes of DBS in patients with PD were diverse, ranging from 4.3 to 34% (see Supplementary Table). This variation may be explained by the fact that DBS was performed in different countries or on patients of different races. However, only one article has been reported in Korea (13). We, therefore, report a larger number of cohorts from our center. The aims of this study were as follows: (1) to investigate the mortality of bilateral STN DBS in patients with PD, (2) to assess the factors associated with mortality, and (3) to assess the cause of death after DBS.

## Methods

### Participants

We conducted a retrospective longitudinal observational study of patients with DBS. We reviewed the medical records of patients with PD who underwent bilateral STN DBS at the movement disorder center (MDC) at the Seoul National University Hospital (SNUH) between March 2005 and May 2018. A PD diagnosis was made based on the United Kingdom Parkinson's Disease Society Brain Bank Criteria (14), and all patients were examined by experienced movement disorder specialists (BJ and HJK). In total, 269 patients were included in this study. Among them, 10 patients who were referred to other hospitals for reoperation were excluded. One patient was further excluded due to DBS of the ventral intermediate nucleus, and another patient was excluded due to thalamotomy surgery. A total of 257 patients who underwent STN DBS surgery were included. Patients who underwent a second operation at our hospital were included, and the time from symptom onset to surgery or surgery to death was calculated based on the first operation.

### Clinical assessment

Preoperative and postoperative assessments were performed according to the SNUH MDC protocol (15). The Unified Parkinson's Disease Rating Scale (UPDRS) parts I, II, and III (motor subscales) and the Hoehn and Yahr (HY) stages were assessed in on-medication and off-medication states. We also measured axial scores, which is the sum of the following motor subscores from the UPDRS-III: stance (item 27), posture (item 28), postural instability (item 29), and gait (item 30) (16). We defined the presence of UPDRS I and II items (e.g., the presence of hallucination, swallowing difficulty, and falling) by a cutoff value of score 2.

The levodopa equivalent daily dose (LEDD) (17) and levodopa doses were calculated at each visit. A diagnosis of PD dementia was made when a patient met the Movement Disorder Society Task Force clinical criteria for PDD. Preoperative cognitive function was evaluated using the Korean Mini-Mental State Examination (MMSE), and depression scores were evaluated using the Beck Depression Inventory with a cutoff score of 16.

Postoperative evaluations were performed at 3, 6, and 12 months after STN DBS, and annually thereafter. Follow-up evaluations were carried out as inpatient procedures at the MDC, with video recordings and diary entries (15). The primary outcomes were survival status and cause of death. The cause and date of death of each patient were obtained from interviews with caregivers or medical certificates at the last follow-up. The cause of death was categorized based on previous studies (10, 12).

### Statistical analysis

A descriptive analysis was used to examine the DBS group. Demographic characteristics are presented as mean values ± standard deviations (SD) or numbers (%), when applicable. The Kaplan–Meier method was used for a survival analysis. Univariate and Cox regression analyses were used to estimate hazard ratios (HRs) and 95% confidence intervals (CIs). We included sex, age at onset, disease duration at surgery, preoperative body mass index (BMI), depression, UPDRS-III score (off-medication score, on-medication score, on-medication score/stimulation on), axial score, falling score, and HY stage as covariates for Cox regression analysis. The proportional hazards assumption considers that HR for comparison groups is constant over the follow-up period, and the proportional hazards assumption was tested by the time-dependent Cox model, including an interaction term with time. HR varied over time in this model, which violated the proportional hazards assumption for two variables (preoperative falling on-medication score and preoperative depression). Thus, we used time-dependent Cox regression analysis by including interaction terms with time (T_COV_^*^ preoperative falling on-medication score, T_COV_^*^preoperative depression). The other variables satisfied the proportional hazards assumption. SPSS ver. 20 (IBM Corp, Armonk, NY) and R version 4.1.2 (R Foundation for Statistical Computing, Vienna, Austria) were used for all statistical analyses, and a *P*-values of below 0.05 were considered statistically significant.

## Results

Table 1 shows the demographic and baseline clinical characteristics of the patients with PD who underwent DBS surgery. Of the 257 patients, 142 (55.3%) were women. The mean ± SD age of PD onset was 46.9 ± 9.5 years, and the mean disease duration at the time of surgery was 11.5 ± 4.6 years. Preoperative HY stage on medication in all patients except one was under 3. One patient was classified as grade 4 due to diphasic dyskinesia. The mean MMSE score at baseline was 27.3 ± 2.5, and no patients had dementia. 58.8% of patients had depression. The mean ± SD preoperative UPDRS-III motor scores on medication and off medication were 21.1 ± 11.7 and 41.6 ± 14.9, respectively. The mean ± SD preoperative axial symptom score on medication and off medication was 2.9 ± 2.2 and 6.5 ± 3.4, respectively. The mean ± SD levodopa-induced dyskinesia (hour) and wearing off time were 7.2 ± 3.8 and 5.1 ± 4.4, respectively.

**Table 1:** Demographic and baseline clinical characteristics of patients with PD who underwent DBS surgery.

**Variable**	***N* = 257**
Sex	
Women	142 (55.3%)
Men	115 (44.7%)
Age at surgery	58.3 ± 8.2 years
Age of onset	46.9 ± 9.5 years
Disease duration at surgery	11.5 ± 4.6 years
LEDD total preop	1362.8 ± 674.0
HY stage preop on-medication	
≤ 2.5	188 (73.2%)
3	68 (26.5%)
4	1 (0.4%)
MMSE score (*n* = 243)	27.3 ± 2.5
Depression (*n* = 245)	151 (58.8%)
UPDRS-III score preop on-medication	21.1 ± 11.7
UPDRS-III score preop off-medication	41.6 ± 14.9
(*n* = 251)	
Preop axial symptom score	6.5 ± 3.4

PD, Parkinson's disease; DBS, deep brain stimulation; LEDD, levodopa equivalent daily dose; preop, preoperative; HY, Hoehn and Yahr; MMSE, Korean mini-mental state examination; UPDRS, unified Parkinson's disease rating scale.

### Mortality and cause of death

**Figure 1:**
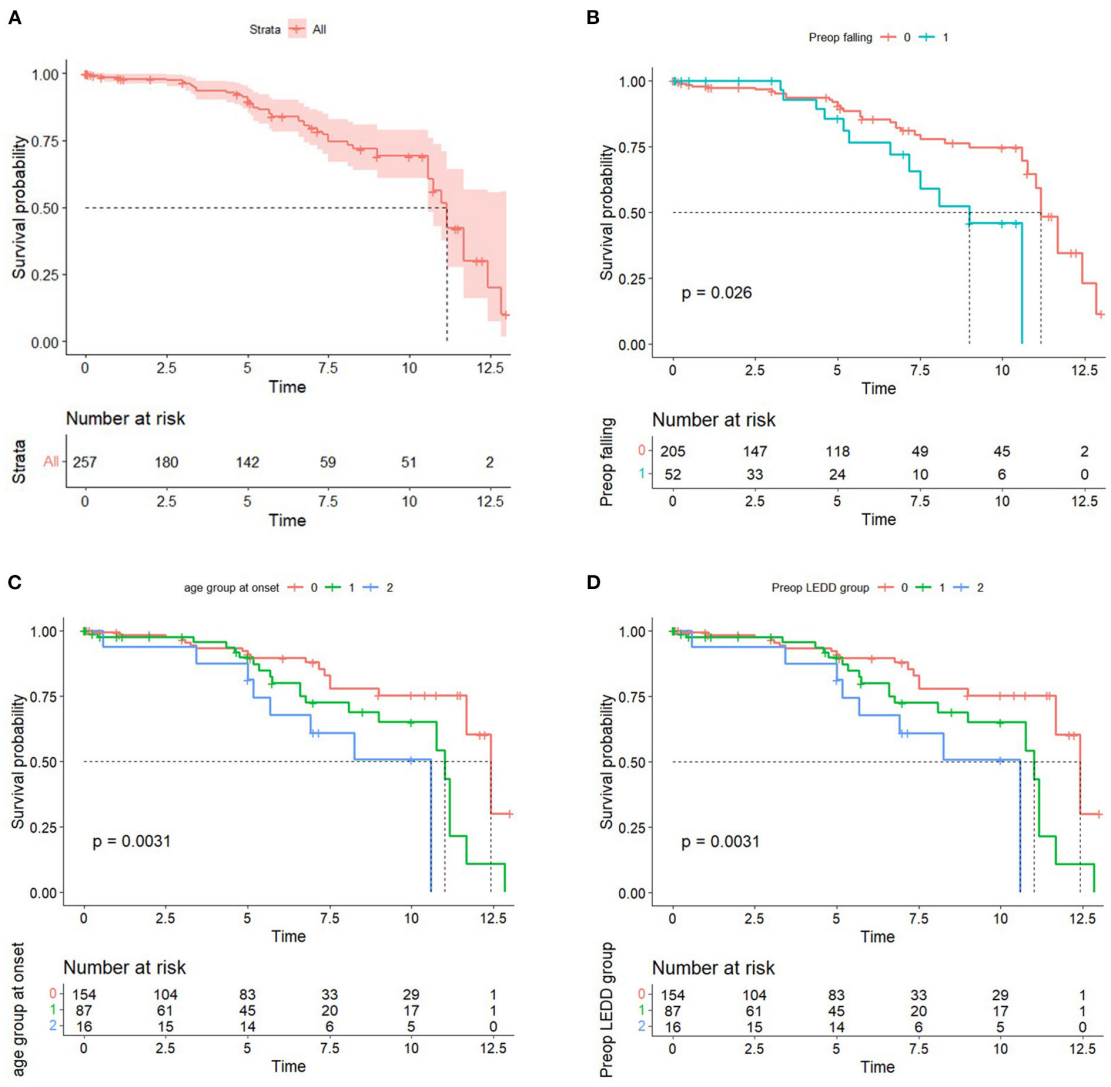
**(A)** Kaplan–Meier curve of the DBS group. **(B)** Kaplan–Meier curve by the presence of preoperative falling on-medication score. **(C)** Kaplan–Meier curve by the age group at onset. 0: <50, 1: 50–59, 2: ≥60. **(D)** Kaplan–Meier curve by the preoperative LEDD group. 0: first tertile, 1: second tertile, and 2: third tertile.

A median follow-up duration was 60 months. One hundred forty-two patients with PD were followed up for 5 years, and 51 patients were followed up for 10 years. Of the 257 patients with PD, 48 patients (18.7%) died, with a median time of death of 11.2 years after surgery. The disease duration was 16.08 years from onset to death. The mean age at death was 67.7 years. For the survival group, the age and duration of PD at the time of the study were 62.2 ± 8.6 and 16.4 ± 5.4 years. Survival curves are shown in Figure 1. The results showed a 1-year survival rate of 98.4%, a 3-year survival rate of 96.8 %, a 5-year survival rate of 89.8%, and a 10-year survival rate of 69.2% for patients with PD who underwent DBS treatment at the study center (Figure 1A). Kaplan–Meier plots illustrated survival probability according to the presence of preoperative falling on medication, age group at onset, and preoperative LEDD group (*P* < 0.05) (Figures 1B–D).

A time-dependent Cox regression analysis revealed that older age of onset (HR = 1.048; 95% CI = 1.005–1.093; *P* = 0.028), higher preoperative total LEDD (HR = 1.001, 95% CI, 1.000–1.001; *P* = 0.038), and the interaction term with Time (T_COV_^*^ preoperative falling on-medication score) (HR = 1.361; 95% CI = 1.001–1.850; *P* = 0.049) were associated with a higher risk of mortality (Table 2). A significant interaction term with time indicates that the effect of preoperative falling on HR changed over time. In detail, the direction of HR (<1 or >1) flip over at ~4 years and then increase more markedly as the follow-up period increase. Figure 1B also supports these results. Sex, preoperative presence of depression, disease duration at surgery, presence of preoperative axial symptoms while on medication, preoperative HY stage, and preoperative BMI were not significantly associated with mortality. However, UPDRS-III preoperative improvement score (UPDRS-III off-medication score minus UPDRS-III on-medication score) and the UPDRS-III postoperative improvement score (preoperative UPDRS-III on-medication score minus UPDRS-III on-medication/stimulation-on score) were not significantly associated with mortality (data not shown). Stimulation parameters, such as amplitude, frequency, and pulse width, were not significantly associated with mortality (data not presented). The causes of death are listed in Table 3. Causes of death were grouped into six categories: (1) progression of PD; (2) psychiatric complications; (3) accidental death; (4) unknown; (5) cancer; and (6) medical complications. Pneumonia was the most common cause of death (26, 54.2%). Four patients (8.3%) died of various cancers, four (8.3%) died by suicide, four (8.3%) died of car accidents, drowning, or falling, two (4.2%) died of other medical complications, and seven (14.6%) died of unknown causes. We also analyzed the causes of death within 5 years after surgery (Table 4). Seven patients (38.9%) died of pneumonia, while four (8.3%) died by suicide 1, 3, 13, and 30 months, respectively, after surgery.

**Table 2:** Associations of clinical features with mortality after deep brain stimulation in patients determined by a time-dependent Cox proportional hazard model with time-dependent variables.

	**Coefficient**	**Hazard ratio (95% CI)**	***P*-value**
Age of onset	0.047	1.048 (1.005–1.093)	0.028
Preoperative total LEDD	0.001	1.001 (1.000–1.001)	0.038
Preoperative falling on-medication score	−1.274	0.280 (0.037–2.098)	0.215
T_COV_^*^preoperative falling on-medication score	0.308	1.361 (1.001–1.850)	0.049

CI, confidence interval; LEDD, levodopa equivalent daily dose Covariates; sex, age at onset, disease duration at surgery, preoperative BMI, depression, T_COV_^*^depression, axial score, falling score, and HY stage.

**Table 3:** Causes of death of patients with Parkinson's disease after undergoing deep brain stimulation.

**Category**	***N* (%)**	**Diagnosis**	***N* (%)**
Disease progression	27 (56.3%)	Pneumonia	26 (54.2%)
		Aging	1 (2.1%)
Unknown	7 (14.6%)	Unknown	6 (12.5%)
		Sudden death	1 (2.1%)
Suicide	4 (8.3%)	Suicide	4 (8.3%)
Accidental death	4 (8.3%)	Car accident	1 (2.1%)
		Drowning	1 (2.1%)
		Falling	2 (4.2%)
Others	2 (4.2%)	Diabetes mellitus complication	1 (2.1%)
		Peptic ulcer perforation	1 (2.1%)
Cancer	4 (8.3%)	Ovarian cancer	1 (2.1%)
		Gastric cancer	1 (2.1%)
		Pancreatic cancer	1 (2.1%)
		Colon cancer	1 (2.1%)
Total	48 (100.0%)		

**Table 4:** Causes of death of patients with Parkinson's disease within 5 years of undergoing deep brain stimulation.

**Category**	***N* (%)**	**Diagnosis**	***N* (%)**
Disease progression	7 (38.9%)	Pneumonia	7 (38.9%)
Unknown	2 (11.1%)	Unknown	2 (11.1%)
Suicide	4 (22.2%)	Suicide	4 (22.2%)
Accidental death	3 (16.7%)	Car accident	1 (5.6%)
		Falling	2 (11.1%)
Cancer	2 (11.1%)	Ovarian cancer	1 (5.6%)
		Gastric cancer	1 (5.6%)
Total	18 (100.0%)		

## Discussion

We studied a large cohort of patients with PD over a mean duration of 5 years after STN DBS surgery. We found that 48 patients with PD (18.7%) of 257 died, with a median time of death of 11.2 years after surgery, and that pneumonia was the most common cause of death. The mortality rate in the present study was 18.7%. The present study was compared with previous studies that reported the mortality of patients with DBS in different countries and groups (Supplementary Table). The mortality outcomes of DBS in patients with PD were diverse, ranging from 4.3 to 34%. This variation may be explained by the fact that DBS was performed in different countries, on patients of different races, with different disease durations at the time of surgery.

Pneumonia was the most common cause of death reported in the present study; many previous studies have also reported pneumonia as a common cause of death (7, 11, 13, 18–20). Cancer, suicide, and falling rates have also been reported in our study. The causes of death showed a pattern similar to that reported in other studies. Lau et al. (16) reported that the most common cause of death was a progression of PD, followed by asphyxiation or aspiration pneumonia. They also reported suicide and death due to cancer. Rocha et al. (20) reported that bacterial pneumonia was the most common cause of death, followed by death due to cancer (9.0%) and suicide (6.6%). Zhang et al. (19) reported that pneumonia and asphyxia were the most common causes of death. Castrioto et al. (7) also reported that aspiration pneumonia was the most common cause of death. A high prevalence of swallowing-related diseases, the severity of which is associated with the duration and severity of PD, has also been reported (21). We considered pneumonia to be a consequence of swallowing-related disorders associated with PD progression. Furthermore, since patients without dementia were initially considered indications for DBS, the onset of dementia was classified as PD progression. Therefore, it was easy to predict that pneumonia would be the most common cause of death.

The suicide rate reported in the present study was higher than that in other studies; however, this is consistent with other studies' reports that suicidal behaviors can occur after STN DBS, especially during the first 3 years (22, 23). Thus, we should regularly assess and treat postoperative depression to prevent suicide, which is a preventable cause of death. Other studies have also reported cancer as a cause of death after STN DBS (5, 11, 12, 16, 18, 24). Thus, by focusing only on the progression of PD, we may neglect other diseases, such as cancer. Regular cancer screening in patients with PD should not be overlooked.

Older age of onset, the occurrence of preoperative falls on medication, and higher total preoperative LEDD were also associated with a higher risk of mortality in the present study. Older age at the time of surgery is a predictor of mortality in patients with DBS, which is in line with the results of previous studies (5, 9). In our study, the presence of preoperative falling on medication, which is considered an axial symptom, was associated with a higher risk of mortality. This is consistent with other studies. Lau et al. (16) suggested that the level of axial disability was the only symptom that significantly predicted death. Higher age of disease onset was reported to be associated with a faster progression rate of axial symptoms and falls in patients with PD (25, 26). Thus, older age at the time of surgery and a high preoperative falling score on medication may indicate high mortality rates. Sex differences did not influence mortality in our patients with DBS, in contrast to other studies (12, 13). Previous studies regarding sex differences did not propose any biological explanation, and thus, further study is needed.

The present study additionally found that a higher total preoperative LEDD was associated with a higher risk of mortality. Furthermore, Fasano et al. (6) reported that the subgroup of patients who developed remarkable worsening of postural stability 8 years after DBS also had marked postural instability at baseline (before surgery) in both the off-medication and on-medication conditions and showed a significantly greater intake of antiparkinsonian medication at baseline. In light of the results of the present study, it can be assumed that the group with a high baseline LEDD had more postural instability, which influenced mortality.

The present study had several strengths. Compared with other similar long-term studies, our study examined one of the largest cohorts, followed up at the same DBS center over a longer period of time. Patients were followed by the same neurosurgeons and neurologists. We were able to contact all patients by telephone or in-person, so none were lost to follow-up, leading to more valid conclusions. However, this study also had several limitations. First, this was a single-center study conducted in South Korea; thus, these results do not represent the entire Korean population. Second, whether DBS could affect the course of PD is a debatable issue, but we do not have control data. Third, we were not able to estimate total electrical energy delivered for DBS frequency due to the unavailability of impedance. Finally, there is no genetic and clinical information such as comorbidity burden and dementia conversion in patients with PD who underwent STN DBS surgery.

In conclusion, this study highlighted the mortality outcomes of STN DBS in patients with PD; older age of PD onset, preoperative falling scores on-medication may be associated with a higher risk of mortality.

## Data availability statement

The raw data supporting the conclusions of this article will be made available by the authors, without undue reservation.

## Ethics statement

The studies involving human participants were reviewed and approved by the Institutional Review Board of Seoul National University Hospital (IRB No.1901-142-1005). Written informed consent for participation was not required for this study in accordance with the national legislation and the institutional requirements.

## Author contributions

AhrK and BJ were involved in the conception and design of the work. H-JK, AryK, and YK recruited participants and collected the data. Data analysis and interpretation was primarily conducted by AhrK, with guidance and advice offered by AhwK, JO, HP, and SP. AhrK wrote the manuscript. BJ and SP commented on and reviewed the manuscript. All authors contributed to the article and approved the submitted version.

## References

[1] Krack P Batir A Van Blercom N Chabardes S Fraix V Ardouin C. Five-year follow-up of bilateral stimulation of the subthalamic nucleus in advanced Parkinson's disease. N Engl J Med. (2003) 349:1925–34. 10.1056/NEJMoa035275 14614167

[2] Deuschl G Schade-Brittinger C Krack P Volkmann J Schäfer H Bötzel K. A randomized trial of deep-brain stimulation for Parkinson's disease. N Engl J Med. (2006) 355:896–908. 10.1056/NEJMoa060281 16943402

[3] Moro E Lozano AM Pollak P Agid Y Rehncrona S Volkmann J. Long-term results of a multicenter study on subthalamic and pallidal stimulation in Parkinson's disease. Mov Disord. (2010) 25:578–86. 10.1002/mds.22735 20213817

[4] Rodriguez-Oroz MC Obeso JA Lang AE Houeto JL Pollak P Rehncrona S. Bilateral deep brain stimulation in Parkinson's disease: a multicentre study with 4 years follow-up. Brain J Neurol. (2005) 128(Pt 10):2240–9. 10.1093/brain/awh571 15975946

[5] Wider C Pollo C Bloch J Burkhard PR Vingerhoets FJ. Long-term outcome of 50 consecutive Parkinson's disease patients treated with subthalamic deep brain stimulation. Parkinsonism Relat Disord. (2008) 14:114–9. 10.1016/j.parkreldis.2007.06.012 17822940

[6] Fasano A Romito LM Daniele A Piano C Zinno M Bentivoglio AR. Motor and cognitive outcome in patients with Parkinson's disease 8 years after subthalamic implants. Brain J Neurol. (2010) 133:2664–76. 10.1093/brain/awq221 20802207

[7] Castrioto A Lozano AM Poon YY Lang AE Fallis M Moro E. Ten-year outcome of subthalamic stimulation in Parkinson disease: a blinded evaluation. Arch Neurol. (2011) 68:1550–6. 10.1001/archneurol.2011.182 21825213

[8] Bove F Mulas D Cavallieri F Castrioto A Chabardès S Meoni S. Long-term outcomes (15 years) after subthalamic nucleus deep brain stimulation in patients with Parkinson disease. Neurology. (2021) 97:e254–62. 10.1212/WNL.0000000000012246 34078713

[9] Toft M Lilleeng B Ramm-Pettersen J Skogseid IM Gundersen V Gerdts R. Long-term efficacy and mortality in Parkinson's disease patients treated with subthalamic stimulation. Movement Disord. (2011) 26:1931–4. 10.1002/mds.23817 21656853

[10] Schüpbach MW Welter ML Bonnet AM Elbaz A Grossardt BR Mesnage V. Mortality in patients with Parkinson's disease treated by stimulation of the subthalamic nucleus. Mov Disord. (2007) 22:257–61. 10.1002/mds.21264 17149702

[11] Rocha S Monteiro A Linhares P Chamadoira C Basto MA Reis C. Long-term mortality analysis in Parkinson's disease treated with deep brain stimulation. Parkinsons Dis. (2014) 2014:717041. 10.1155/2014/717041 24772365 PMC3960527

[12] Bang Henriksen M Johnsen EL Sunde N Vase A Gjelstrup MC Østergaard K. Surviving 10 years with deep brain stimulation for Parkinson's disease: a follow-up of 79 patients. Eur J Neurol. (2016) 23:53–61. 10.1111/ene.12614 25492023

[13] Ryu HS Kim MS You S Kim MJ Kim YJ Kim J. Mortality of advanced Parkinson's disease patients treated with deep brain stimulation surgery. J Neurol Sci. (2016) 369:230–5. 10.1016/j.jns.2016.08.041 27653896

[14] Hughes AJ Daniel SE Kilford L Lees AJ. Accuracy of clinical diagnosis of idiopathic Parkinson's disease: a clinico-pathological study of 100 cases. J Neurol Neurosurg Psychiatry. (1992) 55:181–4. 1564476 10.1136/jnnp.55.3.181 PMC1014720

[15] Lee JY Han JH Kim HJ Jeon BS Kim DG Paek SH. STN DBS of advanced Parkinson's disease experienced in a specialized monitoring unit with a prospective protocol. J Korean Neurosurg Soc. (2008) 44:26–35. 10.3340/jkns.2008.44.1.26 19096653 PMC2588284

[16] Lau B Meier N Serra G Czernecki V Schuepbach M Navarro S. Axial symptoms predict mortality in patients with Parkinson disease and subthalamic stimulation. Neurology. (2019) 92:e2559–e70. 10.1212/WNL.0000000000007562 31043471 PMC6556086

[17] Thobois S. Proposed dose equivalence for rapid switch between dopamine receptor agonists in Parkinson's disease: A review of the literature. Clin Ther. (2006) 28:1–12. 10.1016/j.clinthera.2005.12.003 16490575

[18] Merola A Rizzi L Artusi CA Zibetti M Rizzone MG Romagnolo A. Subthalamic deep brain stimulation: clinical and neuropsychological outcomes in mild cognitive impaired parkinsonian patients. J Neurol. (2014) 261:1745–51. 10.1007/s00415-014-7414-8 24952619

[19] Zhang J Wang T Zhang C-C Zeljic K Zhan S Sun B-M. The safety issues and hardware-related complications of deep brain stimulation therapy: a single-center retrospective analysis of 478 patients with Parkinson's disease. Clin Interv Aging. (2017) 12:923–8. 10.2147/CIA.S130882 28652714 PMC5472429

[20] Rocha AL Oliveira A Sousa C Monteiro P Rosas MJ Vaz R. Long term mortality of patients with Parkinson's disease treated with deep brain stimulation in a reference center. Clin Neurol Neurosurg. (2021) 202:106486. 10.1016/j.clineuro.2021.106486 33493881

[21] Kanna SV Bhanu K. A simple bedside test to assess the swallowing dysfunction in Parkinson's disease. Ann Indian Acad Neurol. (2014) 17:62–5. 10.4103/0972-2327.128556 24753662 PMC3992772

[22] Giannini G Francois M Lhommée E Polosan M Schmitt E Fraix V. Suicide and suicide attempts after subthalamic nucleus stimulation in Parkinson disease. Neurology. (2019) 93:e97–e105. 10.1212/WNL.0000000000007665 31101738

[23] Voon V Krack P Lang AE Lozano AM Dujardin K Schüpbach M. A multicentre study on suicide outcomes following subthalamic stimulation for Parkinson's disease. Brain J Neurol. (2008) 131:2720–8. 10.1093/brain/awn214 18941146 PMC2724899

[24] Weaver FM Stroupe KT Smith B Gonzalez B Huo Z Cao L. Survival in patients with Parkinson's disease after deep brain stimulation or medical management. Mov Disord. (2017) 32:1756–63. 10.1002/mds.27235 29150873

[25] Kempster PA Williams DR Selikhova M Holton J Revesz T Lees AJ. Patterns of levodopa response in Parkinson's disease: a clinico-pathological study. Brain J Neurol. (2007) 130:2123–8. 10.1093/brain/awm142 17586867

[26] de Lau LM Verbaan D Marinus J van Hilten JJ. Survival in Parkinson's disease. Relation with motor and non-motor features Parkinsonism. Relat Disord. (2014) 20:613–6. 10.1016/j.parkreldis.2014.02.030 24679900

