# Supplementary material for: The mortality of patients with Parkinson's disease with deep brain stimulation
Source: Front Neurol. 2023 Jan 16;13:1099862. doi: 10.3389/fneur.2022.1099862 (PMC9885091; doi:10.3389/fneur.2022.1099862)
Supplement: Supplementary file 1 [file Table_1.docx]

Supplementary table. Reported rates and causes of mortality after DBS in PD patients

| **Series** | **Country, year** | **Total no. of DBS patients** | **No. (%) of deceased patients** | **Follow-up period** | **Mean age of PD onset** | **Mean age at surgery** | **Mean PD duration at the time of surgery** | **Gender** | **Causes of death** | **Target site** |
| --- | --- | --- | --- | --- | --- | --- | --- | --- | --- | --- |
| Krack et al.(1) | France, 2003 | 49 | 3 (6.1%) | 5 years | NA | 55.0 ± 7.5 | 14.6 ± 5.0 | M:24/W:25 | 1 intracerebral hemorrhage; 1 myocardial infarction; 1 suicide | STN |
| Rodriguez-Oroz et al.(2) | Multicenter, 2005 | 69 | 3 (4.3%) | 4 years | NA | 59.8 ± 9.8(STN)  55.8 ± 9.4(GPi) | 15.4 ± 6.3  15.4 ± 6.2 | M:25/W:24 (STN)  M:13/W:7 (GPi) | 1 cancer;  1 myocardial infarction;  1 unknown | STN 49  GPi 20 |
| Moro et al. (3) | Multicenter, 2010 | 51 | 10 (19.6%) | 5–6 years | NA | 59.3 ± 1.6(STN)  56.0 ± 2.1(GPi) | 15.3 ± 1.1(STN)  15.1 ± 1.5(GPi) | M:17/W:18 (STN)  M:11/W:5 (GPi) | NA | STN 35  GPi 16 |
| Schüpbach et al. (4) | France, 2007 | 171 | 16 (9.4%) | 41 months  (7–101 months) | 44 (13-61) | 57 (27-75) | 15 (8-28)  14 (4-29) | M:103 (62%)  W:68 (38%) | 6 sudden deaths;  4 PD progression;  4 unrelated to PD;  2 psychiatric complications | STN |
| Wider et al.(5) | Switzerland, 2008 | 50 | 17 (34%) | 5 years | NA | 64.9 ± 7.6 | 14.4 ± 4.9 | M:30  W:20 | 7 infections;  1 cancer;  1 myocardial infarction;  2 pulmonary embolisms;  1 anaphylactic shock;  3 suicides | STN |
| Fasano et al. (6) | Italy, 2010 | 32 (total) | 3 (9.4%) | 8 years | NA | 56.9 ± 7.3 | 14.0 ± 5.5 | M:18  W:14 | cancer;  intraocular melanoma;  pulmonary embolism | STN |
| Toft et al.(7) | Norway, 2011 | 144 | 12 (8.3%) | 1–7 years  Mean 3.3years | NA | 60.3 ± 7.8 | 11.0 ± 4.8 | M:93 (65%)  W:62 (35%) | 2 surgical complications;  2 suicides;  8 complications unrelated to the surgery | STN |
| Castrioto et al.(8) | Canada, 2011 | 41 (total) | 12 (29.3%) | 10 years | 39.6 ± 6.6  (full follow-up) | 52.9 ± 7.9 | 13.4 ± 4.8 | NA | 3 aspiration pneumonia;  2 sepsis;  1 cancer | STN |
| Zibetti et al.(9) | Italy,2011 | 47 (total) | 10 (21.3%) | 9 years | 44.9±7.4 | 61.4 ± 6.0 | 16.4 ± 4.9 | M: 30  W: 17 | NA | STN |
| Rocha et al.(10) | Portugal, 2014 | 184 | 15 (8.15%) | 10 years | 47 ± 9 | 60 ± 8 | 14 | M:113 (61%)  W:71 (39%) | 4 pneumonias;  2 myocardial infarctions  2 hemorrhagic strokes;  cancer; suicide | STN |
| Merola et al.(11) | Italy, 2014 | 174  (134/40) | 26 (15%)  Normal cognition (11.9%)  MCI (25%) | >5 years | 46.40 ± 7.30 | 60.31 ± 6.72 | 13.91 ± 4.75 | M:104 (59.8 %)  W:70 (40.2 %) | 11 pneumonias;  4 sepsis;  3 myocardial infarctions;  3 cancers | STN |
| Ngoga et al.(12) | United Kingdom, 2014 | 106:41  (Surgery:  medial cohort) | 18 (17%) | 10 years | NA | 60 (53-63) | 11 (8.8-13.0) | M:76 (71.7%)  W:30 (28.3%) | 5 PD progression;  4 cardiovascular diseases;  3 gastrointestinal diseases;  2 respiratory diseases | STN |
| Lilleeng et al.(13) | Norway, 2014 | 81:90  (Surgery:  medical cohort) | NA | 7.9 years | NA | 61 ± 7 | NA | M: 65%  W: 35% | NA | STN |
| Ryu et al.(14) | South Korea, 2016 | 158 | 27 (17.1%) | 12 years | 46.0 ± 10.0 | 57.1 ± 8.9 | 16.9 ± 6.1 | M:55 (34.8%)  W:103 (65.2%) | 7 pneumonias;  5 sudden deaths;  3 dementia;  2 suicides | STN 122  GPi 15 |
| Bang Henriksen et al.(15) | Denmark, 2016 | 79 | 24 (30.1%) | 10 years | 43.8 ± 9.2 | 59.7 ± 7.7 | 15.7 ±6.0 | M:52 (66%)  W:27 (34%) | 6 cancers;  4 PD progression;  3 pneumonias | STN |
| Weaver et al.(16) | USA, 2017 | 611:611  (Surgery:  medical cohort) | 168 (27.5%)  214 (35%) | 6.3 years (mean survival) | NA | 69.2 ± 7.4 | NA | NA | 72 PD  13 heart disease;  9 cancers;  3 pneumonias | NA |
| Zhang et al.(17) | China, 2017 | 478 | 41 (8.6%) | 3–16 years | 51.5 ± 15.3 | 57.5 ± 11.0 | NA | M:325 (63%)  W:190 (37%) | 13 pneumonias;  8 asphyxias;  6 organ failure | STN 477  GPi 38 |
| Scelzo et al.(18) | Italy, 2019 | 91/91 (  Surgery:  medical cohort) | 1 (1.1%) | 3.4 years | NA | 60.3 | 11.6 | M:59 (65%)  W:32 (35%) | 1 car accident | STN 87  GPi 4 |
| Lau(19) | France, 2019 | 143 | 41 (28.7%) | 12 years | 43.0 ± 8.4 | 56.3 ± 8.4 | 13.4 ± 5.5 | M:63 %  W:37% | 18 PD progression;  7 choking or aspiration pneumonia;  5 cancers | STN |
| Rocha(20) | Portugal, 2021 | 346 | 62 (17.9%) | 7 ± 4 years  (1–17) | 48 ± 8 | 60 ± 7 | 14 ± 6 | M: 208  W: 138 | 15 bacterial pneumonias;  8 dementia with PD;  4 acute myocardial infarctions;  4 suicides | NA |
| The present study | South Korea | 257 | 48 (18.7%) | 5 years (median) | 46.9 ± 9.5 | 58.3 ± 8.2 | 11.5 ± 4.6 | M: 115 (44.7%)  W: 142 (55.3%) | 24 pneumonias;  8 unknowns;  4 suicides;  4 cancers;  2 medicals complications | STN |

Note. DBS: deep brain stimulation; no: number; MCI: mild cognitive impairment; NA: not available; M: men; W: women; PD: Parkinson’s disease; STN: subthalamic nucleus; GPi: globus pallidus interna

**Supplementary Reference**

1. Krack P, Batir A, Van Blercom N, Chabardes S, Fraix V, Ardouin C, et al. Five-year follow-up of bilateral stimulation of the subthalamic nucleus in advanced Parkinson's disease. N Engl J Med. 2003;349(20):1925-34.

2. Rodriguez-Oroz MC, Obeso JA, Lang AE, Houeto JL, Pollak P, Rehncrona S, et al. Bilateral deep brain stimulation in Parkinson's disease: a multicentre study with 4 years follow-up. Brain. 2005;128(Pt 10):2240-9.

3. Moro E, Lozano AM, Pollak P, Agid Y, Rehncrona S, Volkmann J, et al. Long-term results of a multicenter study on subthalamic and pallidal stimulation in Parkinson's disease. Mov Disord. 2010;25(5):578-86.

4. Schüpbach MW, Welter ML, Bonnet AM, Elbaz A, Grossardt BR, Mesnage V, et al. Mortality in patients with Parkinson's disease treated by stimulation of the subthalamic nucleus. Mov Disord. 2007;22(2):257-61.

5. Wider C, Pollo C, Bloch J, Burkhard PR, Vingerhoets FJ. Long-term outcome of 50 consecutive Parkinson's disease patients treated with subthalamic deep brain stimulation. Parkinsonism Relat Disord. 2008;14(2):114-9.

6. Fasano A, Romito LM, Daniele A, Piano C, Zinno M, Bentivoglio AR, et al. Motor and cognitive outcome in patients with Parkinson's disease 8 years after subthalamic implants. Brain. 2010;133(9):2664-76.

7. Toft M, Lilleeng B, Ramm-Pettersen J, Skogseid IM, Gundersen V, Gerdts R, et al. Long-term efficacy and mortality in Parkinson's disease patients treated with subthalamic stimulation. Movement Disorders. 2011;26(10):1931-4.

8. Castrioto A, Lozano AM, Poon YY, Lang AE, Fallis M, Moro E. Ten-year outcome of subthalamic stimulation in Parkinson disease: a blinded evaluation. Archives of neurology. 2011;68(12):1550-6.

9. Zibetti M, Merola A, Rizzi L, Ricchi V, Angrisano S, Azzaro C, et al. Beyond nine years of continuous subthalamic nucleus deep brain stimulation in Parkinson's disease. Mov Disord. 2011;26(13):2327-34.

10. Rocha S, Monteiro A, Linhares P, Chamadoira C, Basto MA, Reis C, et al. Long-term mortality analysis in Parkinson's disease treated with deep brain stimulation. Parkinsons Dis. 2014;2014:717041.

11. Merola A, Rizzi L, Artusi CA, Zibetti M, Rizzone MG, Romagnolo A, et al. Subthalamic deep brain stimulation: clinical and neuropsychological outcomes in mild cognitive impaired parkinsonian patients. Journal of neurology. 2014;261(9):1745-51.

12. Ngoga D, Mitchell R, Kausar J, Hodson J, Harries A, Pall H. Deep brain stimulation improves survival in severe Parkinson's disease. Journal of neurology, neurosurgery, and psychiatry. 2014;85(1):17-22.

13. Lilleeng B, Brønnick K, Toft M, Dietrichs E, Larsen JP. Progression and survival in Parkinson's disease with subthalamic nucleus stimulation. Acta Neurologica Scandinavica. 2014;130(5):292-8.

14. Ryu HS, Kim MS, You S, Kim MJ, Kim YJ, Kim J, et al. Mortality of advanced Parkinson's disease patients treated with deep brain stimulation surgery. J Neurol Sci. 2016;369:230-5.

15. Bang Henriksen M, Johnsen EL, Sunde N, Vase A, Gjelstrup MC, Østergaard K. Surviving 10 years with deep brain stimulation for Parkinson's disease--a follow-up of 79 patients. Eur J Neurol. 2016;23(1):53-61.

16. Weaver FM, Stroupe KT, Smith B, Gonzalez B, Huo Z, Cao L, et al. Survival in patients with Parkinson's disease after deep brain stimulation or medical management. Mov Disord. 2017;32(12):1756-63.

17. Zhang J, Wang T, Zhang C-C, Zeljic K, Zhan S, Sun B-M, et al. The safety issues and hardware-related complications of deep brain stimulation therapy: a single-center retrospective analysis of 478 patients with Parkinson's disease. Clin Interv Aging. 2017;12:923-8.

18. Scelzo E, Beghi E, Rosa M, Angrisano S, Antonini A, Bagella C, et al. Deep brain stimulation in Parkinson's disease: A multicentric, long-term, observational pilot study. J Neurol Sci. 2019;405:116411.

19. Lau B, Meier N, Serra G, Czernecki V, Schuepbach M, Navarro S, et al. Axial symptoms predict mortality in patients with Parkinson disease and subthalamic stimulation. Neurology. 2019;92(22):e2559-e70.

20. Rocha AL, Oliveira A, Sousa C, Monteiro P, Rosas MJ, Vaz R. Long term mortality of patients with Parkinson's disease treated with deep brain stimulation in a reference center. Clin Neurol Neurosurg. 2021;202:106486.
